# The promoter polymorphism -232C/G of the PCK1 gene is associated with type 2 diabetes in a UK-resident South Asian population

**DOI:** 10.1186/1471-2350-10-83

**Published:** 2009-09-02

**Authors:** Simon D Rees, Abigail C Britten, Srikanth Bellary, J Paul O'Hare, Sudhesh Kumar, Anthony H Barnett, M Ann Kelly

**Affiliations:** 1 College of Medical and Dental Sciences, University of Birmingham, Birmingham, UK; 2 Heart of England NHS Foundation Trust, Birmingham, UK; 3 Warwick Medical School, University of Warwick, Coventry, UK

## Abstract

**Background:**

The *PCK1 *gene, encoding cytosolic phosphoenolpyruvate carboxykinase (PEPCK-C), has previously been implicated as a candidate gene for type 2 diabetes (T2D) susceptibility. Rodent models demonstrate that over-expression of *Pck1 *can result in T2D development and a single nucleotide polymorphism (SNP) in the promoter region of human *PCK1 *(-232C/G) has exhibited significant association with the disease in several cohorts. Within the UK-resident South Asian population, T2D is 4 to 6 times more common than in indigenous white Caucasians. Despite this, few studies have reported on the genetic susceptibility to T2D in this ethnic group and none of these has investigated the possible effect of *PCK1 *variants. We therefore aimed to investigate the association between common variants of the *PCK1 *gene and T2D in a UK-resident South Asian population of Punjabi ancestry, originating predominantly from the Mirpur area of Azad Kashmir, Pakistan.

**Methods:**

We used TaqMan assays to genotype five tagSNPs covering the *PCK1 *gene, including the -232C/G variant, in 903 subjects with T2D and 471 normoglycaemic controls.

**Results:**

Of the variants studied, only the minor allele (G) of the -232C/G SNP demonstrated a significant association with T2D, displaying an OR of 1.21 (95% CI: 1.03 - 1.42, *p *= 0.019).

**Conclusion:**

This study is the first to investigate the association between variants of the *PCK1 *gene and T2D in South Asians. Our results suggest that the -232C/G promoter polymorphism confers susceptibility to T2D in this ethnic group.

**Trial Registration:**

**UKADS Trial Registration**: ISRCTN38297969

## Background

Cytosolic phosphoenolpyruvate carboxykinase (PEPCK-C), encoded by the *PCK1 *gene in humans, is an enzyme centrally involved in gluconeogenesis, glyceroneogenesis and cataplerosis. Normal expression of *PCK1 *is under hormonal control, regulated at the transcriptional level by both activators, such as glucagon, and inhibitors, such as insulin. The metabolic functions of PEPCK-C advocate the *PCK1 *gene as a strong candidate for conferring susceptibility to type 2 diabetes (T2D), a theory that is supported by the effects of gene modulation in mouse and rat models. These include over-expression of PEPCK-C resulting in insulin resistance and T2D [1].

Genetic studies have previously implicated the region of chromosome 20q in which *PCK1 *lies in T2D susceptibility [2,3]. Cao *et al. *[4] reported on the discovery of a single nucleotide polymorphism (SNP) in the promoter region of *PCK1 *(-232C/G) that was associated with T2D in Canadian Caucasian and Oji-Cree cohorts (odds ratio (OR) = 2.8, 95% CI 1.7 - 4.7, *p *= 4 × 10^-5 ^and OR = 1.9, 95% CI 1.2 - 3.0, *p *= 9 × 10^-3 ^respectively). In addition, luciferase reporter assays demonstrated that the -232G allele increased expression of *PCK1 *compared to the -232C allele in multiple cell lines, with no down regulation by insulin. As with many genetic association studies investigating polygenic diseases, attempts to replicate the association between *PCK1 *and T2D have produced mixed results. A haplotype of *PCK1 *variants was shown to confer risk of developing the disease in a Korean population (OR not given, *p *= 6 × 10^-3^) [5], and a screen of 134 candidate susceptibility SNPs showed that the -232C/G SNP was a risk factor for T2D in a Finnish cohort (OR = 1.27, 95% CI 1.02 - 1.57, *p *= 0.031) [6]. A recent study reported that multiple *PCK1 *variants are associated with T2D in a Chinese population [7]. The authors of this paper reported -232C as a risk allele, although its association with the disease fell short of statistical significance (OR = 1.24, 95% CI 1.00 - 1.55, *p *= 0.057). Studies investigating other populations, however, have found no evidence for an association between *PCK1 *variants and T2D [8,9].

Within the UK, T2D is 4 to 6 times more common in the South Asian population compared to the indigenous white Caucasian population [10]. Over 10% of South Asian adults will develop the disease and yet only a small number of investigations have reported on the genetic susceptibility to T2D in this ethnic group. None of these studies has looked into the possible effects of the *PCK1 *gene. We therefore aimed to investigate the association between common variants of the *PCK1 *gene and T2D in a UK-resident South Asian population of Punjabi ancestry.

## Methods

Type 2 diabetic subjects (N = 903) were recruited to the United Kingdom Asian Diabetes Study (UKADS), a multiple risk factor intervention trial investigating the impact of a culturally-sensitive, enhanced diabetes care package on the risk of cardiovascular disease in South Asian type 2 diabetes patients living in Birmingham and Coventry, UK [11]. All subjects were of Punjabi ancestry, confirmed over three generations, and originated predominantly from the Mirpur area of Azad Kashmir, Pakistan. Ethnically-matched normoglycaemic control subjects (N = 471) were recruited from the same geographical areas through community screening. Normal glucose tolerance was defined as fasting plasma glucose <6 mmol/l and 2 hr plasma glucose <7.8 mmol/l on a 75 g OGTT. Where OGTT was not feasible, normal glucose tolerance was defined as random blood glucose <7 mmol/l. Venous blood was collected from each subject after obtaining informed consent and genomic DNA extracted using an adaptation of the Nucleon^® ^protocol (Nucleon Biosciences, Coatbridge, UK). The study was approved by the Birmingham East, North and Solihull Research Ethics Committee.

### SNP selection and genotyping

In addition to investigating the -232C/G promoter polymorphism (rs2071023), we also utilised Haploview 3.2 [12] to tag SNPs within the entire *PCK1 *gene. To do this we used data from the CEPH (CEU) HapMap samples (Utah residents with ancestry from northern and western Europe)[13], as this population was the closest proxy for our South Asian population available on the HapMap at the time tag SNPs were chosen. Our criteria for tagging SNPs were r^2 ^≥ 0.7 and a minor allele frequency (MAF) ≥ 0.15, using pairwise tagging only. This resulted in four extra SNPs (rs6070157, rs2070756, rs2179706 and rs1042531) for analysis. All SNPs were genotyped using TaqMan SNP Genotyping assays (Applied Biosystems, Warrington, UK) and fluorescence was measured using an ABI 7900 sequence detection system (Applied Biosystems).

### Statistical analyses

Genotype frequencies for each SNP were checked for Hardy-Weinberg equilibrium using a chi square goodness-of-fit test. Pairwise linkage disequilibrium (LD) between SNPs was estimated using Haploview version 3.2. Variants were tested for association with type 2 diabetes using logistic regression, assuming an additive genetic model. Possible confounding variables (BMI, gender, family history of T2D) were initially included in the logistic regression as covariates. Haplotype analyses were performed using Haploview version 3.2. Association between genotypes and continuous variables was tested using analysis of variance (ANOVA). The significance of the relationship between OR and minimum age threshold was determined using linear regression. All of the above statistical analyses were implemented in SPSS version 13.0 (SPSS Inc, Chicago IL). Power calculations were performed using Genetic Power Calculator [14].

## Results

The clinical characteristics of the subjects in our study are shown in Table 1. Age of diagnosis, HDL cholesterol and HbA_1c _data were available for subjects with diabetes only, whereas BMI, waist circumference and blood pressure measurements were available for both the diabetic group and a maximum of 279 subjects from the control group. Genotypes of the studied *PCK1 *SNPs were not significantly associated with any clinical, biochemical or morphological characteristic measured (Table 2).

**Table 1:** Clinical characteristics of subjects studied

	**Diabetic subjects**		**Control subjects**	
	**Male**	**Female**	**Male**	**Female**

**n**	494	409	230	241

**Age at study (years)**	57.1 ± 11.9	56.7 ± 12.3	57.1 ± 11.8	53.0 ± 11.6

**Age at diagnosis (years)**	49.8 ± 11.7	49.3 ± 12.3	NA^a^	NA^a^

**BMI (kg/m^2^)**	27.4 ± 4.1	30.0 ± 4.9	27.1 ± 4.1	28.9 ± 5.3

**Waist circumference (cm)^c^**	100.9 ± 10.1	104.2 ± 11.0	100.5 ± 11.2	99.0 ± 13.8

**Systolic blood pressure (mm Hg)^c^**	141.4 ± 19.3	138.9 ± 22.5	139.5 ± 19.6	131.1 ± 20.2

**Diastolic blood pressure (mm Hg)**	84.5 ± 12.6	84.1 ± 12.0	86.0 ± 11.6	82.9 ± 12.1

**HDL cholesterol (mmol/l)**	1.3 ± 0.6	1.3 ± 0.4	ND^b^	ND^b^

**HbA_1c _(%)**	8.3 ± 2.0	8.3 ± 1.7	ND^b^	ND^b^

Data are expressed as means ± SD. ^a^NA = not applicable. ^b^ND = not determined. ^c ^= significant difference between diabetic and control groups (t-test; *p *< 0.01).

**Table 2:** Clinical characteristics of subjects studied

	**Diabetic subjects**			**Control subjects**		
**rs2071023**	**CC**	**CG**	**GG**	**CC**	**CG**	**GG**

BMI (kg/m^2^)	28.8 ± 4.6	28.4 ± 4.7	28.9 ± 4.7	28.5 ± 5.2	27.7 ± 4.8	28.3 ± 4.1
Waist circumference (cm)	102.8 ± 10.5	102.1 ± 11.0	103.0 ± 10.0	100.1 ± 13.4	98.8 ± 11.5	101.5 ± 13.7
Systolic blood pressure (mm Hg)	140.6 ± 20.6	139.7 ± 20.1	141.1 ± 22.7	134.4 ± 19.3	135.2 ± 20.5	136.3 ± 22.2
Diastolic blood pressure (mm Hg)	84.0 ± 11.5	84.2 ± 13.2	85.2 ± 11.8	84.8 ± 11.2	84.2 ± 12.2	84.1 ± 12.7
HDL cholesterol (mmol/l)	1.3 ± 0.5	1.3 ± 0.5	1.3 ± 0.5	ND	ND	ND
HbA_1c _(%)	8.3 ± 1.8	8.2 ± 1.8	8.4 ± 2.1	ND	ND	ND

**rs6070157**	**CC**	**CT**	**TT**	**CC**	**CT**	**TT**

BMI (kg/m^2^)	28.6 ± 4.7	28.6 ± 4.8	28.0 ± 3.4	28.0 ± 4.7	28.1 ± 5.0	28.7 ± 5.3
Waist circumference (cm)	102.5 ± 10.3	102.4 ± 11.0	102.0 ± 13.4	99.5 ± 13.0	99.0 ± 12.1	103.8 ± 10.4
Systolic blood pressure (mm Hg)	140.1 ± 20.9	140.5 ± 20.5	139.3 ± 19.9	133.9 ± 20.5	136.6 ± 20.1	139.6 ± 20.3
Diastolic blood pressure (mm Hg)	84.3 ± 11.7	84.6 ± 14.4	83.9 ± 7.9	83.6 ± 12.0	85.5 ± 12.3	86.4 ± 9.9
HDL cholesterol (mmol/l)	1.3 ± 0.5	1.3 ± 0.5	1.2 ± 0.3	ND	ND	ND
HbA_1c _(%)	8.4 ± 2.0	8.2 ± 1.7	8.0 ± 1.6	ND	ND	ND

**rs2070756**	**CC**	**CT**	**TT**	**CC**	**CT**	**TT**

BMI (kg/m^2^)	28.5 ± 4.5	28.7 ± 4.8	28.3 ± 4.5	28.0 ± 4.9	27.9 ± 4.8	29.1 ± 5.0
Waist circumference (cm)	102.2 ± 10.7	102.8 ± 10.6	102.3 ± 10.2	99.4 ± 12.3	99.3 ± 12.9	102.7 ± 13.8
Systolic blood pressure (mm Hg)	139.6 ± 20.5	141.8 ± 21.6	138.7 ± 19.1	134.7 ± 20.3	136.9 ± 20.7	132.8 ± 19.5
Diastolic blood pressure (mm Hg)	84.7 ± 13.0	84.3 ± 12.0	83.5 ± 10.4	84.8 ± 11.5	84.0 ± 12.0	84.1 ± 14.2
HDL cholesterol (mmol/l)	1.3 ± 0.5	1.3 ± 0.5	1.3 ± 0.4	ND	ND	ND
HbA_1c _(%)	8.2 ± 1.9	8.3 ± 1.8	8.5 ± 2.1	ND	ND	ND

**rs2179706**	**CC**	**CT**	**TT**	**CC**	**CT**	**TT**

BMI (kg/m^2^)	28.7 ± 4.9	28.5 ± 4.6	28.5 ± 4.4	28.5 ± 4.6	27.8 ± 4.8	27.8 ± 5.1
Waist circumference (cm)	103.0 ± 10.5	102.0 ± 10.7	102.4 ± 10.8	102.6 ± 13.8	98.1 ± 11.7	98.3 ± 12.1
Systolic blood pressure (mm Hg)	141.2 ± 20.8	140.2 ± 21.7	139.2 ± 18.7	134.4 ± 21.0	135.0 ± 19.6	137.0 ± 21.0
Diastolic blood pressure (mm Hg)	84.8 ± 11.1	83.9 ± 13.9	85.3 ± 10.5	83.5 ± 12.2	85.1 ± 12.0	84.7 ± 11.0
HDL cholesterol (mmol/l)	1.3 ± 0.5	1.3 ± 0.5	1.2 ± 0.3	ND	ND	ND
HbA_1c _(%)	8.4 ± 2.0	8.3 ± 1.8	8.0 ± 1.7	ND	ND	ND

**rs1042531**	**TT**	**GT**	**GG**	**TT**	**GT**	**GG**

BMI (kg/m^2^)	28.4 ± 4.4	28.9 ± 4.8	28.4 ± 5.2	28.2 ± 5.1	27.5 ± 4.6	29.1 ± 4.0
Waist circumference (cm)	102.2 ± 10.2	103.1 ± 11.0	101.3 ± 11.2	99.7 ± 13.0	99.1 ± 11.3	101.5 ± 17.1
Systolic blood pressure (mm Hg)	139.2 ± 20.3	140.2 ± 20.6	144.5 ± 22.8	135.9 ± 20.0	136.1 ± 20.2	128.3 ± 22.2
Diastolic blood pressure (mm Hg)	83.6 ± 11.0	84.8 ± 14.1	85.8 ± 10.4	85.4 ± 11.6	84.0 ± 12.2	82.4 ± 12.5
HDL cholesterol (mmol/l)	1.3 ± 0.4	1.3 ± 0.5	1.3 ± 0.6	ND	ND	ND
HbA_1c _(%)	8.2 ± 1.8	8.3 ± 1.8	8.5 ± 2.4	ND	ND	ND

Genotypes of the studied *PCK1 *variants were not significantly associated with any measured clinical characteristic (ANOVA; *p *> 0.05 for all tests). Data are expressed as means ± SD. ND = not determined.

**Figure 1:**
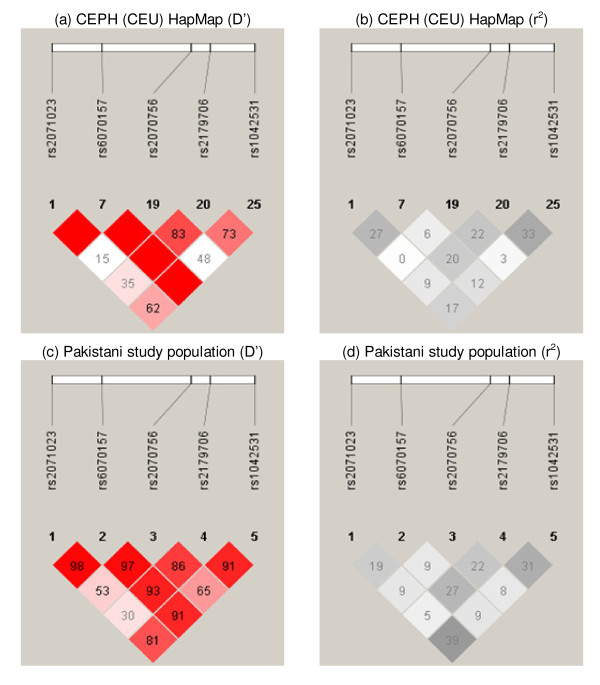
**Pairwise linkage disequilibrium (LD) in CEPH (CEU) HapMap samples and the Pakistani study population**. LD expressed as D' and r^2 ^for the CEPH (CEU) HapMap samples and the Pakistani study population. Blank squares equate to a D' value of 1.

LD patterns (Figure 1) and allele frequencies for all variants were generally similar to those seen in the CEPH (CEU) HapMap samples (CEU allele frequencies: rs2071023 (G) - 0.52, rs6070157 (T) - 0.21, rs2070756 (T) - 0.25, rs2179706 (T) - 0.52, rs1042531 (G) - 0.31). Genotyping success rate was ≥ 97.5% for all SNPs studied. Approximately 15% of all individuals were re-genotyped for the estimation of error rate, which was <1% for all variants. All SNPs conformed to Hardy-Weinberg equilibrium with the exception of rs6070157 (*p *= 0.01). As the error rate for this SNP was zero and it displayed no significant association with any variable measured, no further action was taken to investigate this anomaly.

Of the variants studied, only the minor allele of rs2071023 displayed a significant association with T2D, with an OR of 1.21 (95% CI 1.03 - 1.42, *p *= 0.019; Table 3). A number of clinical and morphological characteristics (gender, BMI, family history of T2D) were included as covariates in our initial analyses, but had no qualitative effect on the observed association and so were excluded from the final model. As statistical power was low for all variants (≤ 72% power to reject a false negative for all variants), we cannot categorically state that the other SNPs studied confer no susceptibility to T2D. We have previously confirmed that variants of *TCF7L2 *confer susceptibility to T2D in this population [15]. Genotype of the *TCF7L2 *SNP rs7903146 was therefore included in the logistic regression model as a covariate, but had no effect on the results and so was excluded from the final model.

**Table 3:** Association of *PCK1 *variants with type 2 diabetes among UK-resident South Asians

**SNP**	**Gene Region**	**Allele**	**Diabetic subjects F^a^**	**Control subjects F^a^**	**Genotype**	**Diabetic subjects n (%)^b^**	**Control subjects n (%)^b^**	**Allelic OR^c ^(95% CI)**
rs2071023	Promoter	C	0.53	0.57	CC	243 (0.27)	158 (0.34)	1.21
					CG	447 (0.51)	225 (0.48)	(1.03 - 1.42)
		G	0.47	0.43	GG	195 (0.22)	88 (0.19)	*p *= 0.019

rs6070157	Exon 3	C	0.81	0.79	CC	597 (0.67)	305 (0.65)	0.90
					CT	258 (0.29)	135 (0.29)	(0.74 - 1.08)
		T	0.19	0.21	TT	38 (0.04)	29 (0.06)	*p *= 0.257

rs2070756	Exon 7	C	0.72	0.71	CC	456 (0.52)	246 (0.53)	1.00
					CT	354 (0.40)	171 (0.37)	(0.84 - 1.18)
		T	0.28	0.29	TT	75 (0.08)	47 (0.10)	*p *= 0.965

rs2179706	Intron 8	C	0.58	0.56	CC	298 (0.34)	156 (0.34)	0.92
					CT	424 (0.48)	206 (0.45)	(0.79 - 1.08)
		T	0.42	0.44	TT	156 (0.18)	100 (0.22)	*p *= 0.323

rs1042531	3'UTR	T	0.66	0.68	TT	391 (0.44)	206 (0.45)	1.06
					GT	384 (0.44)	212 (0.46)	(0.89 - 1.25)
		G	0.34	0.32	GG	104 (0.12)	44 (0.10)	*p *= 0.528

^a^F = Allele frequency. ^b^Genotype frequency expressed as number of individuals, n - values in parentheses indicate percentage. ^c^OR = odds ratio, 95% CI = 95% confidence interval. Allelic OR refers to the risk associated with a single copy of the risk allele. All values were calculated using the full dataset, the minimum age of control subjects being 35 years.

Only one haplotype, comprising the rs2071023 G allele and the rs6070157 C allele, was significantly associated with T2D (Haplotype 1; OR = 1.20, *p *= 0.024; Table 4). This was the only haplotype to contain the rs2071023 G allele and the effect on disease risk was similar to that of rs2071023 alone, suggesting that the haplotype association was due solely to the rs2071023 SNP.

**Table 4:** Association of *PCK1 *haplotypes with type 2 diabetes among UK-resident South Asians

**LD Block**	**Haplotype**	**SNP1**	**SNP2**	**SNP3**	**SNP4**	**Diabetic subjects F^a^**	**Control subjects F^a^**	**Odds ratio (OR)**	***p***
1	Haplotype 1	G	C	-	-	0.472	0.427	1.20	0.024
1	Haplotype 2	C	C	-	-	0.341	0.369	0.89	0.150
1	Haplotype 3	C	T	-	-	0.185	0.204	0.89	0.239

2	Haplotype 4	-	-	C	T	0.405	0.417	0.95	0.538
2	Haplotype 5	-	-	C	C	0.311	0.297	1.06	0.475
2	Haplotype 6	-	-	T	C	0.271	0.264	1.03	0.710
2	Haplotype 7	-	-	T	T	0.014	0.021	0.63	0.130

^a^F = Haplotype frequency. All values were calculated using the full dataset, the minimum age of control subjects being 35 years. SNP1 = rs2071023, SNP2 = rs6070157, SNP3 = rs2070756, SNP4 = rs2179706.

Including young control subjects within a case-control analysis can artificially reduce effect size and statistical significance, as it can increase the chance of including subjects who will develop T2D later in life. As we have done previously with *TCF7L2 *[15], we re-analyzed our data using subsets of the control group defined by different minimum age cut-offs. For SNP rs2071023 there was a significant relationship between control-group minimum age cut-off and both OR (r^2 ^= 0.912, *p *= 3.86 × 10^-7^) and statistical significance of the logistic regression test (r^2 ^= 0.838, *p *= 1.12 × 10^-5^), up until maximum statistical significance (minimum *p*-value) was reached at an age cut-off of 47 years. At this age cut-off the effect size of the association had greatly increased (OR = 1.31, 95% CI 1.10 - 1.56, *p *= 2 × 10^-3^), remaining significant even after correcting for multiple testing (Bonferroni correction for testing 5 SNPs, *p *= 0.015). After this age cut-off both relationships began to deteriorate as the number of individuals within the control group was further reduced. It is interesting to note that when an age cut-off of 47 years was applied to the control group, statistical power actually increased to 90% due to the increase in OR, despite a drop in subject numbers.

## Discussion

As previously reported in a number of studies[4,6] our results suggest that the -232C/G SNP (rs2071023) located in the promoter region of the PEPCK-C-encoding *PCK1 *gene is associated with T2D.

It has been suggested that T2D could be caused by either excessive PEPCK-C production in the liver or reduced levels of PEPCK-C in adipose tissue [16]. In addition, from expression analysis of luciferase reporter constructs in multiple cell lines, Cao *et al*. [4] demonstrated that the -232G risk allele resulted in increased basal gene expression when compared to the -232C allele. It is possible, therefore, that the -232C/G polymorphism may confer increased risk of T2D development by increasing *PCK1 *expression in the liver. This would result in an upregulation of gluconeogenesis and increased blood glucose levels. Unfortunately we were not able to investigate the relationship between *PCK1 *genotype and blood glucose levels in this study, as fasting blood glucose data were only available for a small subset of our control subjects. Interestingly, a recent study has shown that liver-specific silencing of *Pck1 *can improve glycaemic control and insulin sensitivity in a T2D mouse model [17], supporting the role of PEPCK-C in T2D pathology and providing a potential therapeutic target for treatment of the disease.

Although the use of our entire cohort resulted in an OR of 1.21, the removal of young control subjects should have increased the validity of the control group. The OR of 1.31 resulting from our reduced dataset, similar to that seen in a Finnish cohort (OR = 1.27) [6], may therefore be a more representative estimate of the true effect size of this SNP, one that is not inconsiderable compared to recently discovered T2D susceptibility variants.

There are a number of limiting factors to be considered when interpreting our results. Firstly, our cohort is limited in size. Secondly, there is the possibility of cryptic relatedness within our cohort as the study subjects were recruited from a relatively small migrant population. In addition to this consanguinity is relatively common in the Mirpuri population. These limitations increase the risk of our result being a false positive. Unfortunately we cannot control for relatedness in our analyses as we do not have the necessary data. The impact of cryptic relatedness on association studies, however, increases with sample size [18], so it may be that our relatively small cohort is to some degree protected from this effect. Furthermore, we have no reason to believe that the degree of relatedness would differ significantly between the diabetic and control groups and so the effect of any cryptic relatedness may be negated.

## Conclusion

This study is the first to investigate the association between variants of the *PCK1 *gene and T2D in South Asians. In agreement with studies in other ethnic groups [4,6], our analyses suggest that the -232C/G promoter SNP (rs2071023) confers susceptibility to T2D. Due to the limitations discussed within this manuscript, however, we cannot exclude the possibility that our findings are a false positive result. We strongly advocate that replication of this association in larger independent South Asian cohorts is needed to confirm the -232C/G polymorphism of *PCK1 *as a true T2D susceptibility variant in this ethnic group.

## Competing interests

None of the authors declare any competing interests. The United Kingdom Asian Diabetes Study received financial support from all companies mentioned in the Acknowledgements.

## Authors' contributions

SDR participated in the genotyping of samples, performed statistical analyses and drafted the manuscript. ACB performed DNA extraction and genotyping. MAK participated in the drafting of the manuscript. All authors contributed to the intellectual content of the paper, and have read and approved the final manuscript.

## Pre-publication history

The pre-publication history for this paper can be accessed here:


